# Decreased Hemithorax in an Adult

**DOI:** 10.31662/jmaj.2025-0030

**Published:** 2025-08-01

**Authors:** Keisuke Watanabe, Takeshi Kaneko

**Affiliations:** 1 Department of Pulmonology, Yokohama City University Graduate School of Medicine, Yokohama, Japan; 2 Current affiliation: Department of Pulmonary Medicine, International University of Health and Welfare Atami Hospital, Atami, Japan

**Keywords:** computed tomography, congenital disorders, unilateral absence of the pulmonary artery

A 55-year-old woman was referred to our hospital owing to dyspnea on exertion and abnormalities on chest images. Her vital signs were unremarkable. Physical examination revealed normal vascular sounds, no murmurs, no jugular vein distention, and no edema. Chest radiography showed decreased size of the right hemithorax, elevation of the right diaphragm, and absence of the right pulmonary artery shadow (Figure 1). Chest computed tomography (CT) showed absence of the right pulmonary artery and right lung volume loss (Figure 2). The echocardiogram showed no congenital heart disease and no pulmonary hypertension. The patient was diagnosed with congenital isolated absence of the right pulmonary artery.

**Figure 1.**
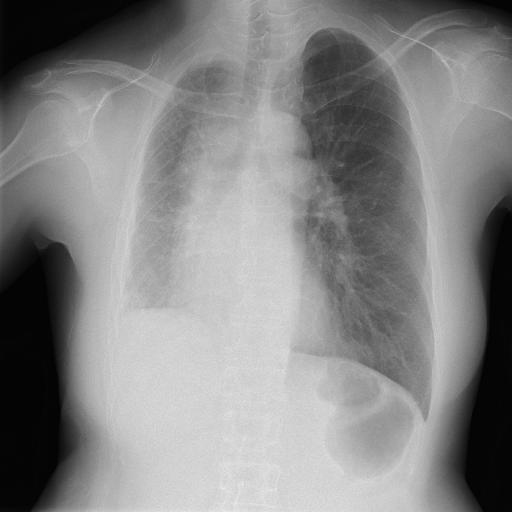
Chest radiography. Chest radiography shows decreased size of the right hemithorax, elevation of the right diaphragm, and absence of the right pulmonary artery shadow.

**Figure 2.**
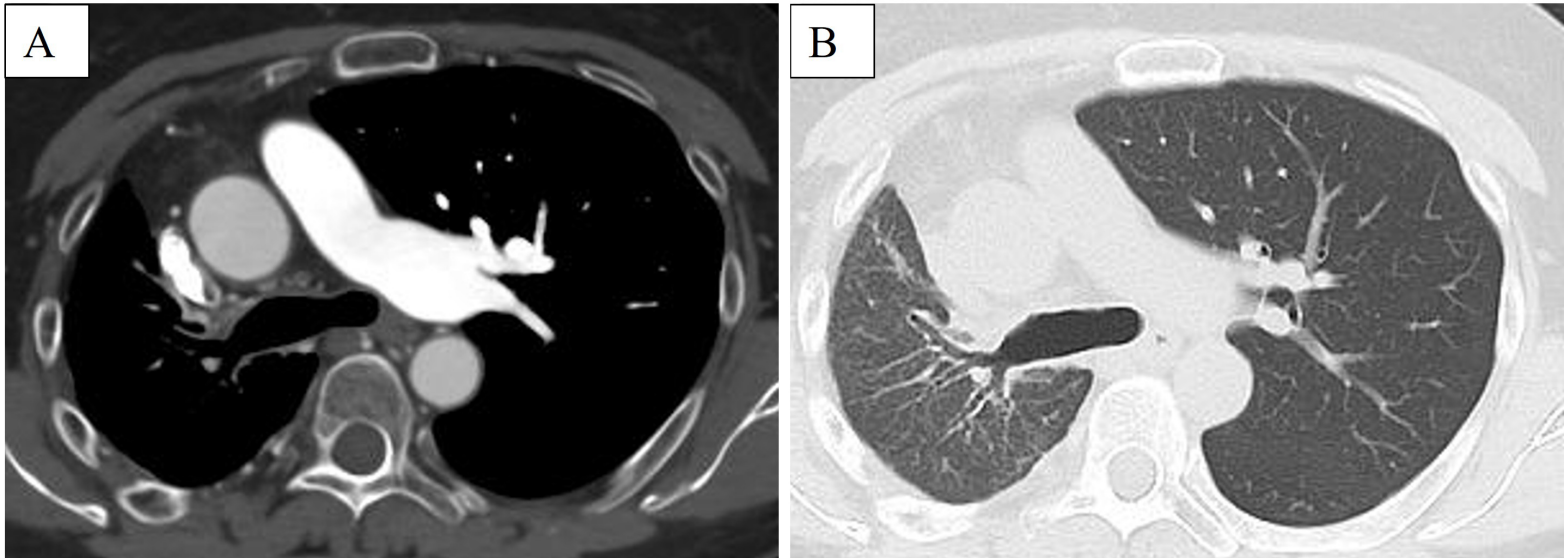
Enhanced chest CT. Chest CT shows absence of the right pulmonary artery and right lung volume loss (A: mediastinal window; B: lung window). CT: computed tomography.

The features of chest radiography in this disease include a decreased size of the affected hemithorax, an absent ipsilateral, and enlarged contralateral pulmonary artery shadow ^(1), (2)^. Chest CT shows a unilateral absence of the pulmonary artery, volume loss of the affected lung, and a systemic-to-pulmonary shunt ^(1), (2)^.

## Article Information

### Conflicts of Interest

None

### Author Contributions

Keisuke Watanabe collected the clinical data and wrote the initial draft of the manuscript. Takeshi Kaneko supervised and edited the manuscript. Both authors read and approved the final manuscript.

### Approval by Institutional Review Board (IRB)

This study was approved by the institutional review board of Yokohama City University Hospital (number 2024-041).

### Informed Consent

Written informed consent was obtained from the patient to publish the case report.

### Declaration of Generative AI and AI-Assisted Technologies in the Writing Process

During the preparation of this work, the authors used ChatGPT to improve readability and language. After using this tool/service, the authors reviewed and edited the content as needed and take full responsibility for the content of the publication.
